# Single-Molecule AFM-Based Force Spectroscopy Reveals VLA-4/VCAM-1 Interactions Underlying Chemokine-Independent B16 Melanoma Cell Arrest

**DOI:** 10.3390/biology15171489

**Published:** 2026-09-02

**Authors:** Robert H. Eibl

**Affiliations:** 1 Institute of Pathology, Technical University of Munich, Trogerstr. 18, 81675 Munich, Germany; robert.eibl@alumni.dkfz.de; 2 Institute of Applied Physics, Ludwig-Maximilians-University of Munich, Amalienstr. 54, 80799 Munich, Germany; 3 Center for NanoScience (CeNS), Schellingstr. 4, 80799 Munich, Germany; 4 Max Planck Institute for Polymer Research, Ackermannweg 10, 55128 Mainz, Germany; 5 I.L. Weissman Laboratory, Department of Pathology, Stanford University, Stanford, CA 94305, USA

**Keywords:** atomic force microscopy (AFM), single-molecule force spectroscopy, VLA-4, VCAM-1, B16 melanoma, cell adhesion, cell migration, metastasis, integrin inhibitor

## Abstract

Integrin-mediated adhesion is essential for leukocyte trafficking and contributes to hematogenous tumor metastasis. We recently showed that metastatic B16 melanoma cells undergo VLA-4-dependent rolling and rapid arrest on endothelial cells under physiological shear flow without classical chemokine-induced integrin activation. However, the molecular basis of this chemokine-independent adhesion remained unresolved. Here, atomic force microscopy (AFM)-based single-molecule force spectroscopy was used to characterize VLA-4-mediated adhesion between living B16 melanoma cells and bEnd.3 endothelial cells at the level of individual receptor–ligand interactions. Rupture-force histograms revealed a dominant population centered at approximately 33 pN, consistent with single VLA-4/VCAM-1 bonds. VLA-4 blockade reduced adhesion frequency, whereas VCAM-1 blockade shifted the rupture-force distribution toward lower forces; both effects support molecular specificity. VLA-4-directed antibody and pharmacological inhibition further indicated that regulation primarily affects bond-formation probability rather than the strength of individual receptor–ligand interactions. Homotypic VLA-4-mediated adhesion between melanoma cells was also detected. These findings provide a functional link between single-molecule receptor interactions and the rolling-to-arrest transition of metastatic melanoma cells under physiological flow. Together with our recent flow-chamber studies, they support a model in which VLA-4/VCAM-1 bond formation contributes to vascular arrest without requiring classical integrin activation, providing molecular insight into an early step of metastatic dissemination.

## Simple Summary

Cancer cells that spread through the bloodstream must first attach to the inner surface of blood vessels before they can invade distant organs. This process resembles the way immune cells leave the circulation, but our recent work showed that metastatic B16 melanoma cells can adhere to blood vessels without the chemokine activation normally required for leukocytes. In this study, we use atomic force microscopy (AFM) to measure the forces between individual adhesion molecules on living melanoma and endothelial cells. These measurements reveal that tumor cell adhesion is mediated by specific VLA-4/VCAM-1 receptor interactions with characteristic single-molecule forces. Blocking either interaction partner reduces adhesion. Together with our recent flow-chamber studies, these findings provide a molecular explanation for how melanoma cells arrest within the vasculature without classical chemokine signaling. Understanding these earliest adhesion events may support the development of therapies aimed at preventing metastatic dissemination.

## 1. Introduction

**Figure 1:**
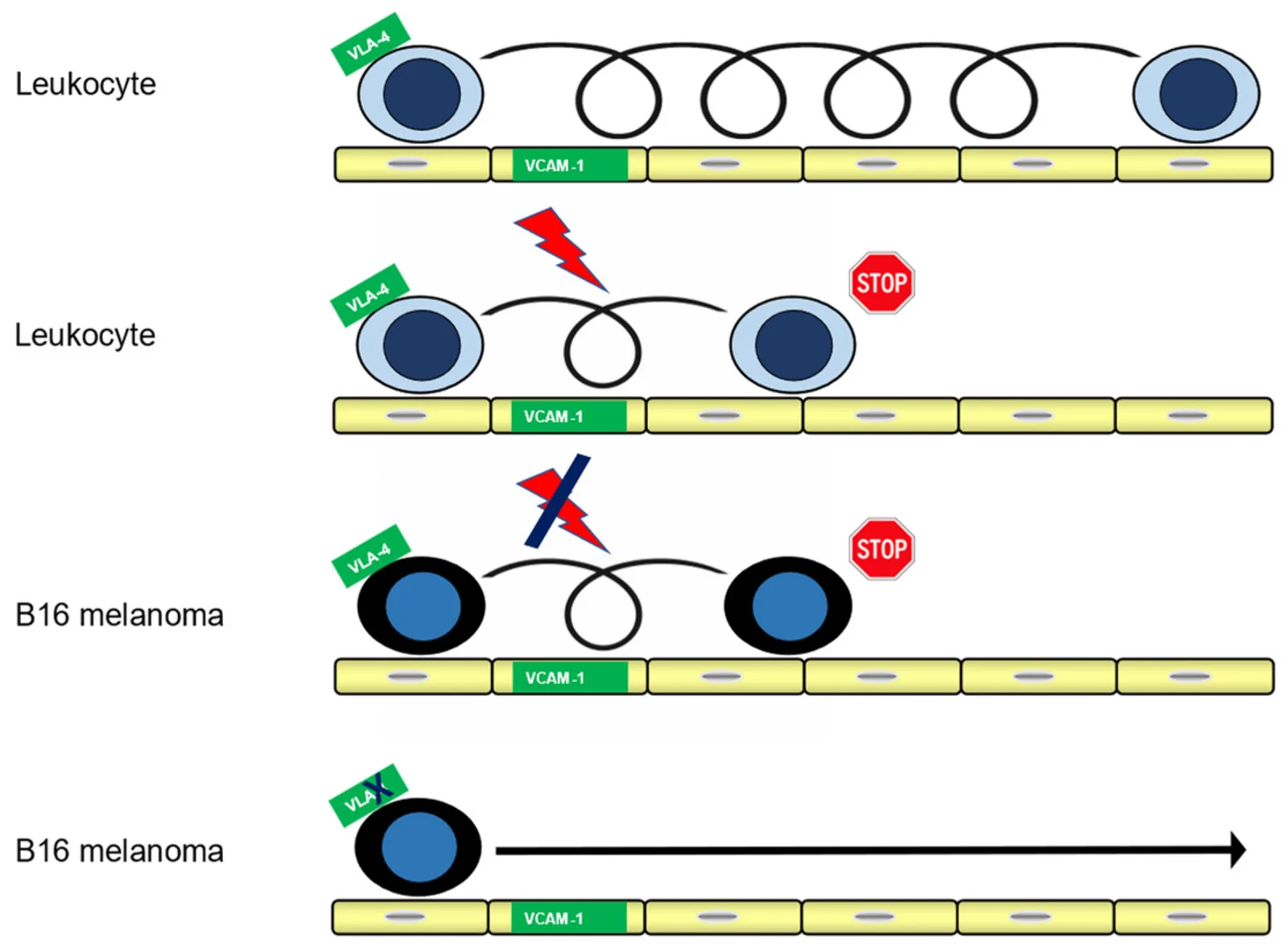
Distinct adhesion mechanisms governing leukocyte versus B16 melanoma rolling-to-arrest transitions. Schematic comparison of leukocyte versus B16 melanoma interactions with VCAM-1 under laminar shear. In leukocytes, rolling is followed by chemokine-dependent Gαi-mediated signaling, which promotes integrin-dependent arrest (PTX-sensitive). In contrast, B16-BL6 melanoma cells roll and undergo rapid arrest in a chemokine-independent, PTX-insensitive manner. Both rolling and arrest are inhibited by VLA-4 blockade. Symbols indicate the presence (lightning) or absence (crossed lightning) of chemokine-dependent signaling. The crossed VLA-4 symbol indicates VLA-4 blockade. The arrow indicates the direction of flow. The schematic summarizes the experimental findings and is not drawn to scale (from Eibl, 2026 [6], with permission).

Metastatic dissemination involves a series of coordinated steps, including vascular arrest of circulating tumor cells, which shares key molecular features with leukocyte adhesion and homing [1]. In leukocytes, this process is classically described by a multistep adhesion cascade consisting of transient rolling followed by integrin-mediated firm arrest, typically requiring chemokine-induced activation [2,3,4]. Integrin VLA-4 (α4β1) plays a central role in this process by mediating both rolling and arrest through interaction with VCAM-1 expressed on activated endothelial cells [5]. While this mechanism is well established in leukocytes, its relevance for tumor cells has remained less clear. Recent work using parallel-plate flow chamber assays has found that B16 melanoma cells exhibit robust VLA-4–dependent rolling and rapid arrest on activated endothelial monolayers under physiological shear conditions [6]. Notably, this behavior occurs independently of chemokine signaling, indicating that tumor cells can bypass canonical Gαi-coupled integrin activation pathways that are typically required in leukocyte adhesion (Figure 1).

**Figure 2:**
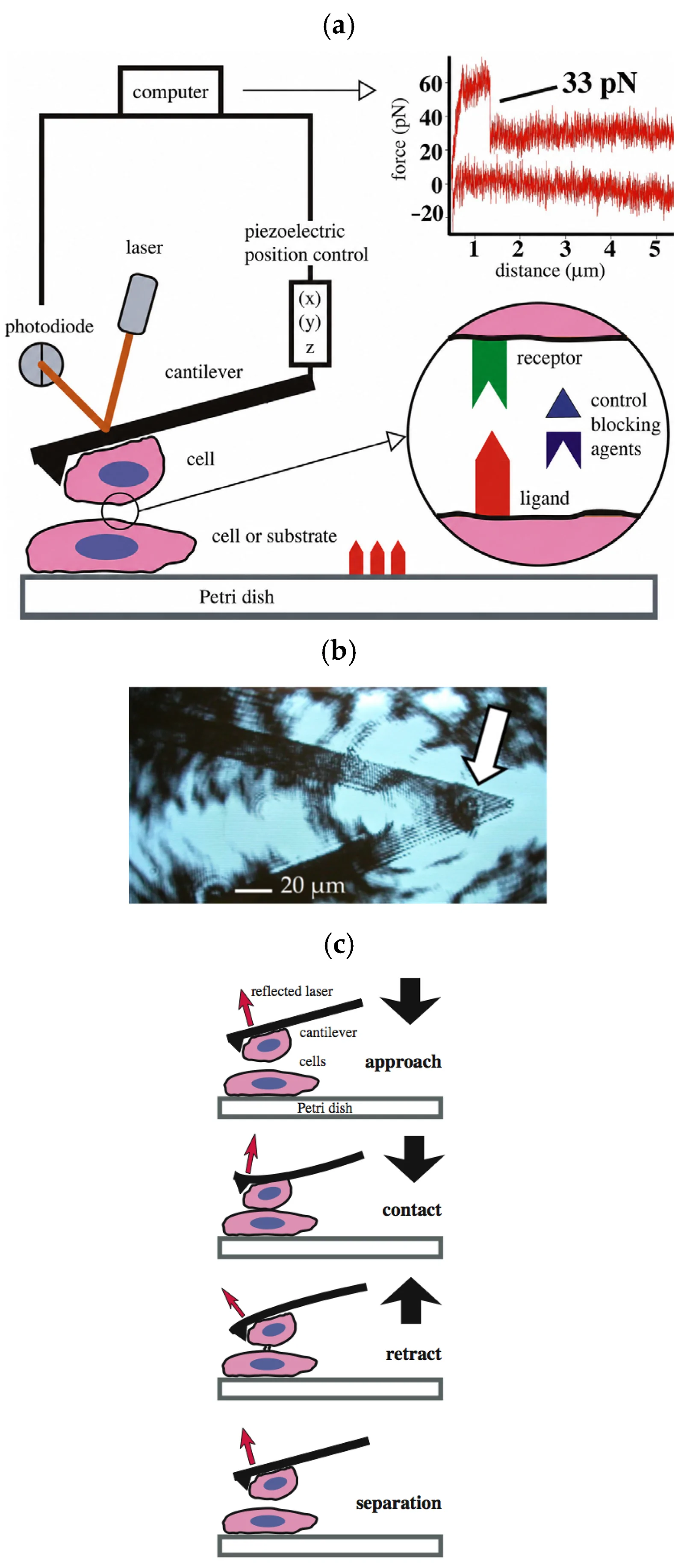
Force measurements. (**a**) Schematic setup of the AFM force-spectroscopy to measure unbinding forces of single receptor–ligand bonds on living cells under physiologic temperature and pH. (**b**) Micrograph of a cell (white arrow) attached to the tip of a cantilever with the laser light from the backside (slightly out of focus for better visibility). (**c**) Scanning steps between two living cells. Objects are not drawn to scale.

These findings suggest that metastatic tumor cells actively engage the vascular endothelium through receptor-mediated mechanisms that are functionally analogous to leukocyte adhesion but physiologically distinct. In particular, the rapid transition from rolling to firm arrest observed for B16 melanoma cells implies that integrin-mediated adhesion can occur without classical affinity upregulation known for leukocytes. While flow-based assays define the macroscopic adhesion phenotype under shear conditions, they do not resolve the molecular interactions underlying these processes. In particular, it remains unclear how individual receptor–ligand bonds contribute to the observed adhesion behavior, whether adhesion is governed by changes in bond strength or bond formation probability, and how these interactions respond to molecular perturbations. Atomic force microscopy (AFM)-based force spectroscopy enables direct measurement of adhesion forces between individual receptor–ligand pairs on living cells under near-physiological conditions [7], including temperature and pH (Figure 2) [8,9]. For physiologically relevant cell-adhesion measurements, receptor-specific interactions should be distinguished from nonspecific adhesion by using defined ligands, counter-receptors, or cellular binding partners [9,10,11]. By controlling contact force and duration, single-molecule conditions can be achieved, allowing discrimination between single and multiple bond interactions [9,10].

In earlier work [8], this approach was used to quantify single VLA-4/VCAM-1 interactions between B16 melanoma cells and activated bEnd.3 endothelial cells, revealing characteristic rupture forces in the range of ~33 pN under defined single-molecule conditions [9]. These studies established for the very first time the feasibility of resolving integrin-mediated adhesion at the single-bond level between living cells under physiologic pH and temperature, and showed that careful control of contact force and duration allows discrimination between single and multiple bond events. However, these initial measurements primarily focused on force quantification and did not address functional regulation, molecular specificity under perturbation, or pharmacological modulation. In the present study, we extend AFM-based single-molecule force spectroscopy to dissect VLA-4-mediated adhesion between B16 melanoma cells and activated bEnd.3 endothelial cells in a functional context. Building on previous flow-based observations and earlier AFM measurements, we aim to establish a direct functional link between adhesion behavior under shear and receptor-level interactions.

## 2. Materials and Methods

### 2.1. Cell Culture and Reagents

Recombinant mouse VCAM-1/CD106 Fc chimera (643-VM), recombinant mouse ICAM-1/CD54 Fc chimera (796-IC), and function-blocking antibodies against mouse VLA-4 (α4β1/CD49d) and VCAM-1/CD106 were obtained from R&D Systems (Minneapolis, MN, USA). A mannose-derived inhibitor was described previously and provided by H. Kessler (compound **11a**; 1 mM) [12].

Murine B16-BL6 melanoma cells were kindly provided by Dr. I.J. Fidler (The University of Texas MD Anderson Cancer Center, Houston, TX, USA; also available as: ATCC CRL-6475; American Type Culture Collection; ATCC, Manassas, VA, USA). This highly metastatic subline was originally selected in vivo from B16-F10 melanoma cells for enhanced invasive and metastatic capacity. Throughout this study, the term B16 refers exclusively to the B16-BL6 subline.

Murine brain endothelial bEnd.3 cells were kindly provided by B. Holzmann (Technical University of Munich, Munich, Germany); the cell line is also available from ATCC (CRL-2299). Cells were maintained in Dulbecco’s Modified Eagle Medium (DMEM; Gibco, Life Technologies, Paisley, UK) supplemented with 10% fetal calf serum (FCS; Gibco, Life Technologies, Paisley, UK), 100 U/mL penicillin, 100 µg/mL streptomycin (Gibco), and 0.1% L-glutamine (Gibco) under standard cell culture conditions (37 °C, humidified atmosphere containing 5% CO_2_).

For AFM measurements, single B16 cells were harvested from subconfluent cultures by short incubation with ethylenediaminetetraacetic acid (EDTA; Sigma, St. Louis, MO, USA) at 4 °C without trypsinization in order to preserve cell-surface adhesion receptors. Cells were concentrated, washed in PBS and resuspended in culture medium containing 20 mM HEPES buffer (Sigma, St. Louis, MO, USA). Confluent bEnd.3 monolayers were activated with 1 µg/mL lipopolysaccharide (LPS; Sigma, St. Louis, MO, USA) for 6–18 h, depending on the experiment. Shorter induction times were used in selected experiments to obtain sufficiently low adhesion probabilities for predominantly single-molecule measurements. Immediately before force measurements, a small number of B16 cells was transferred into the Petri dish on the X-Y-stage of the AFM and a cell was immobilized onto the tip of the cantilever under visual control with the optical microscope from underneath (Figure 2b).

### 2.2. AFM-Based Force Spectroscopy

All AFM-based force spectroscopy measurements were performed under near-physiological conditions (37 °C, pH 7.4), essentially as described previously [8,13], using a custom-built AFM optimized for single-molecule force measurements between living mammalian cells [11]. Cells were attached to the tip of a Si3N4 cantilever (MSCT-AUHW; Park Scientific Instruments, Sunnyvale, CA, USA), which was previously mechanically modified by removing the pyramidal extension to avoid interference with an attached cell and breaking one of the two legs to further reduce the nominal spring constant by half and achieve higher resolution [9]. The spring constant of each cantilever was determined using a thermal noise technique described earlier (about 0.005 N/m) [14]. Functionalization with poly-D-lysine (PDL; 0.1 mg/mL; Sigma, St. Louis, MO, USA) enabled stable cell immobilization while maintaining cell viability during force measurements. A custom-made heating stage under the Petri dish allowed maintenance of physiologic temperature.

Experimental conditions were adjusted to yield adhesion probabilities of approximately 30% or lower. Assuming independent bond formation following Poisson statistics, an adhesion probability of 30% corresponds to a mean number of bonds of λ = −ln(0.70) = 0.357; under this assumption, approximately 83% of adhesion-positive contacts are expected to contain a single bond. Thus, these conditions favor, but do not completely exclude, single-bond interactions [9,10,13]. Importantly, for the analysis of VLA-4/VCAM-1 rupture forces, only force curves containing a single discrete rupture event were included; curves showing two or more rupture steps were excluded in their entirety. The instrument was mounted on an inverted optical microscope (Zeiss Axiomat, Carl Zeiss Inc., Oberkochen, Germany). Under visual control of the optical microscope, the Petri dish was adjusted in the x- and y-axes by hand to identify a single cell of interest and mount it to the cantilever by moving the Petri dish along the z-axis towards the sensor by a piezo-controlled positioning stage with a range of 100 µm. The laser (λ = 655 nm) was focused on the back of the gold-coated cantilever, and the reflected light was detected by a two-segment photodetector.

For a typical force measurement, a B16 melanoma cell adhering to the cantilever was positioned above an activated bEnd.3 cell and then lowered until the sensor detected the preselected contact force (typically 30 pN). After contact was established for a certain time (200 ms), the cell was lifted up by the piezo-actuator at a velocity of 1.3–2.3 µm/s while the adhesion force was monitored in a force–distance plot. Rupture-force histograms were generated from the deadhesion step of individual force–distance curves. Igor Pro 4.0 (WaveMetrics Inc., Lake Oswego, OR, USA) software was customized for automated analysis of force–distance curves and generation of rupture-force histograms. Representative histograms were selected because overlaying pooled datasets from independent experiments would obscure the characteristic single-molecule rupture-force distributions.

### 2.3. Statistical Analysis

All experiments were independently repeated at least three times with comparable results. Representative force–distance curves and representative rupture-force histograms are shown. Histograms summarize rupture forces obtained from individual AFM force curves recorded during representative experiments. Similar qualitative changes in rupture-force distributions were observed in all independent experiments. Owing to the exploratory and functional nature of the study, statistical comparisons of histogram distributions were not performed.

### 2.4. Use of Generative Artificial Intelligence in Manuscript Preparation

ChatGPT (OpenAI, GPT-5.6 Thinking) was used during manuscript preparation for language editing, structural revision, and assistance in drafting selected passages. No generative AI tool was used for experimental design, data generation, data analysis, or modification of figures.

## 3. Results

### 3.1. Single-Molecule Adhesion Events Between B16 Melanoma and Immobilized VCAM-1

**Figure 3:**
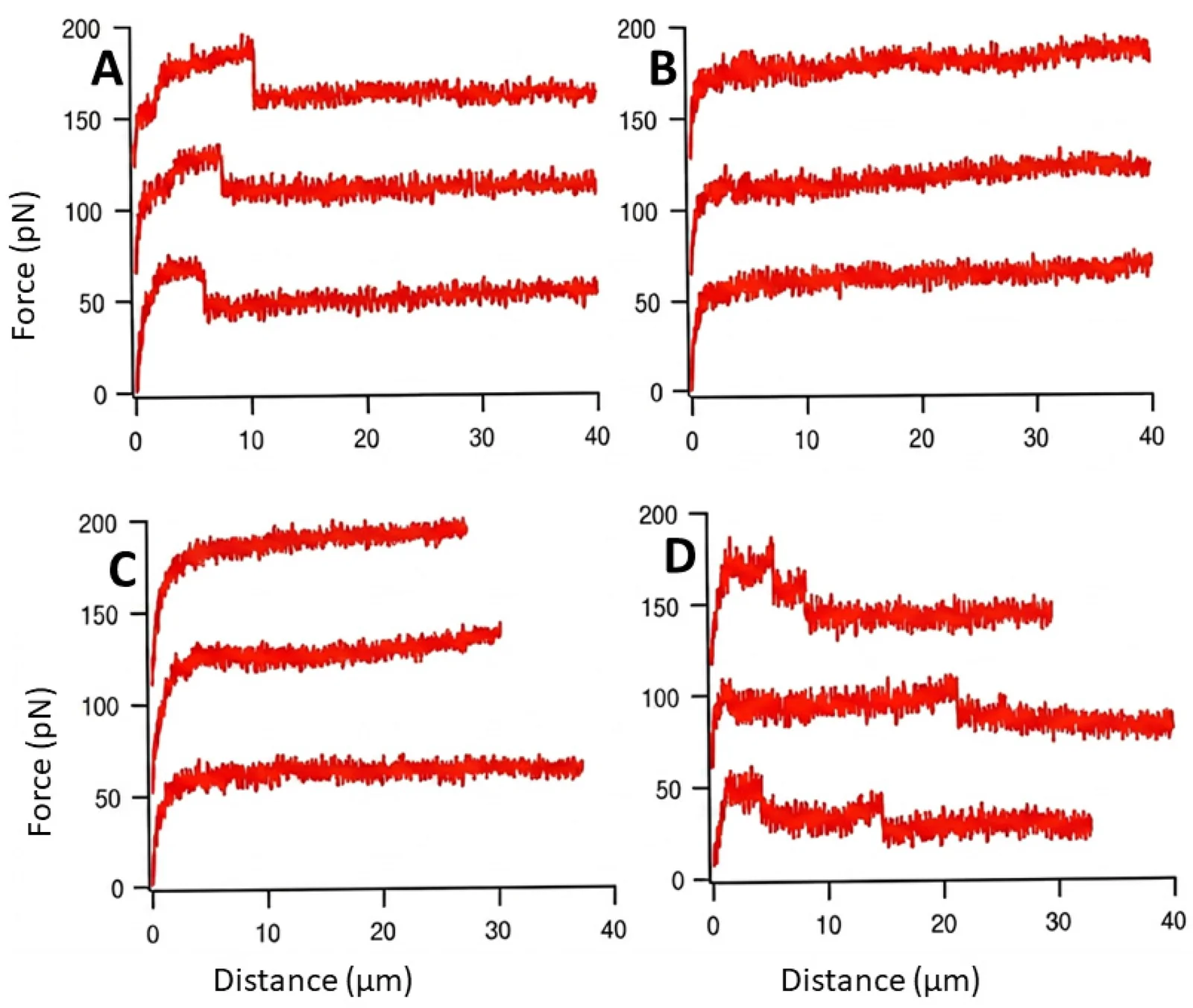
AFM measurements of B16 cells against immobilized substrate. Under near physiologic conditions (pH 7.4; 37 °C), a living B16 melanoma cell was positioned onto the tip of a cantilever and then probed against different immobilized substrates. (**A**) VCAM-1 Fc fusion protein; single ruptures can be detected. (**B**) Blocking with anti-VCAM-1 antibodies prevents single ruptures. (**C**) ICAM-1, no ruptures detected (negative control). (**D**) Fibronectin; single and multiple ruptures detected. Shown are representative backward scans from independent experiments. *n* = 3.

Under conditions optimized for single-molecule detection, AFM-based force spectroscopy measurements between individual B16 melanoma cells and different substrates revealed discrete rupture events on immobilized VCAM-1 Fc fusion protein at the so-called single-molecule level (Figure 3A), which can be blocked with anti-VCAM-1 (Figure 3B). As a negative control, probing against immobilized ICAM-1 Fc fusion protein did not show any typical ruptures (Figure 3C). Probing against fibronectin, which is another ligand for VLA-4, also allowed detection of rupture events.

### 3.2. Single-Molecule Adhesion Events Between B16 Melanoma and Endothelial Cells

**Figure 4:**
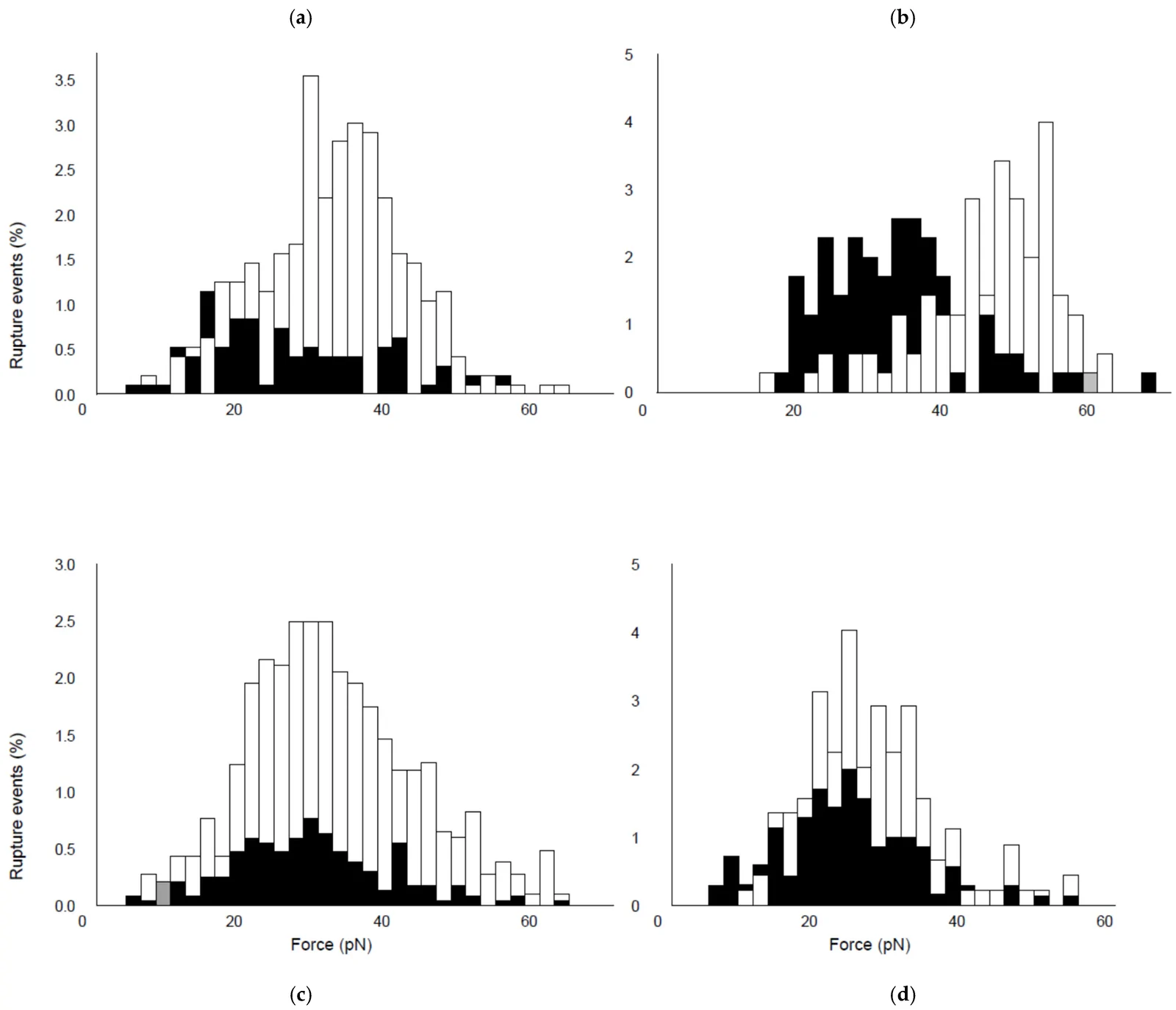
AFM-based rupture-force histograms demonstrating the molecular specificity of VLA-4-mediated adhesion in living B16 melanoma cells. Histograms summarize rupture forces obtained from individual AFM force–distance curves under conditions optimized for predominantly single-molecule measurements. White bars indicate the reference distribution and black bars the corresponding inhibited distribution; the smaller bar is displayed in the foreground to preserve visibility of both distributions. Where both values are identical, a single gray bar is shown. (**a**) Adhesion of B16 melanoma cells to LPS-activated bEnd.3 endothelial cells. Function-blocking anti-CD49d antibodies markedly reduced the frequency of rupture events, consistent with inhibition of VLA-4-dependent adhesion. (**b**) Effect of function-blocking anti-CD106 (VCAM-1) antibodies on B16–bEnd.3 adhesion. VCAM-1 blockade shifted the rupture-force distribution toward lower forces, while interactions around 30 pN remained detectable. (**c**) Pharmacological inhibition of VLA-4-mediated adhesion using the mannose-derived α4β1 integrin antagonist compound **11a** (MW 366), which markedly reduced the frequency of detectable rupture events. (**d**) Homotypic adhesion between two living B16 melanoma cells. Anti-CD49d antibodies reduced the frequency of rupture events, indicating that VLA-4 contributes to B16–B16 adhesion.

AFM-based force spectroscopy measurements between B16 melanoma cells and activated bEnd.3 endothelial cells revealed discrete rupture events consistent with receptor-mediated adhesion. Under conditions optimized for single-molecule detection, rupture force histograms showed a dominant population in the range of ~20–50 pN (Figure 4a), consistent with previously reported single-bond interactions (~33 pN average, SD 12 pN) (Figure 4a–c) [8]. Importantly, the observed rupture-force range is consistent with the forces expected for single VLA-4/VCAM-1 interactions and thus provides a molecular basis for the adhesion events observed under flow conditions. These results support the notion that the rolling-to-arrest transition described previously can be mediated by discrete receptor–ligand interactions at the single-molecule level.

### 3.3. VLA-4 Blockade Supports Molecular Specificity

Blocking of VLA-4 with function-blocking anti-CD49d antibodies altered the adhesion-force distribution between B16 melanoma and activated bEnd.3 endothelial cells (Figure 4a). Compared with untreated measurements, antibody treatment reduced the number of detectable adhesion events, supporting the idea that a major fraction of the interactions was dependent on VLA-4. These findings support VLA-4 as a major contributor to the measured single-molecule adhesion events between B16 melanoma and endothelial cells.

### 3.4. VCAM-1 Blockade Reduces Adhesion Forces

To further investigate the molecular specificity of B16–endothelial cell adhesion, VCAM-1 was blocked with function-blocking anti-CD106 antibodies during force measurements between B16 melanoma cells and LPS-activated bEnd.3 endothelial cells. Comparison of the rupture-force distributions before and after VCAM-1 blockade showed a marked shift toward lower forces, while interactions around 30 pN remained detectable (Figure 4b). These findings further support the involvement of VCAM-1 in VLA-4-mediated B16–endothelial cell adhesion [6].

### 3.5. Pharmacological Inhibition Reduces Adhesion Frequency

To determine whether VLA-4-mediated adhesion could also be modulated pharmacologically, B16–bEnd.3 interactions were measured in the presence of a low-molecular-weight, mannose-derived peptidomimetic α4β1 integrin inhibitor (compound **11a**; MW 366) [12]. Addition of the inhibitor reduced the number of detectable rupture events (Figure 4c). Similar reductions were observed in three independent experiments. Pharmacological inhibition of VLA-4 primarily reduced the probability of VLA-4-mediated bond formation rather than the force of the remaining interactions. These findings provide independent pharmacological evidence for the contribution of VLA-4 to B16 melanoma–endothelial cell adhesion.

### 3.6. Homotypic VLA-4-Mediated Adhesion in Melanoma Cells

To investigate whether VLA-4 also contributes to interactions between melanoma cells, AFM force measurements were performed between two living B16 cells. Under control conditions, discrete rupture events were detected over a characteristic force range (Figure 4d), which was lower than between B16 and activated bEnd.3 cells. Blocking VLA-4 with anti-CD49d antibodies reduced the frequency of detectable adhesion events. These findings show that VLA-4 also contributes to homotypic B16–B16 cell adhesion. The residual low-force interactions observed after VLA-4 blockade may represent VLA-4-independent interactions; however, their molecular identity was not determined in the present experiments.

## 4. Discussion

The present study provides molecular insight into the receptor-level interactions underlying the chemokine-independent transition from rolling to firm arrest previously observed under physiological shear flow [6]. In our recent parallel-plate flow chamber study, metastatic B16 melanoma cells were shown to undergo VLA-4-dependent rolling followed by rapid firm arrest on activated endothelial cells without requiring the classical chemokine-mediated integrin activation that is considered essential for leukocyte arrest [2,3,4,15].

While these experiments established the functional role of VLA-4/VCAM-1 interactions under flow, they did not resolve the molecular interactions responsible for this behavior. The AFM-based single-molecule measurements presented here bridge this gap by demonstrating that the observed adhesion phenotype can be explained by discrete receptor-level interactions between VLA-4 and VCAM-1 measured directly between living cells under near-physiological conditions, including physiologic temperature and pH.

The present work extends our previous AFM study describing single VLA-4/VCAM-1 interactions between living B16 melanoma and endothelial cells [8]. That earlier work established the experimental conditions required for reliable single-molecule measurements and identified a characteristic rupture force of approximately 33 pN for individual VLA-4/VCAM-1 receptor pairs. Its primary focus was methodological and biophysical, demonstrating—for the first time, to the best of our knowledge—the feasibility of resolving individual adhesion receptor–ligand interactions between two living mammalian cells under near-physiological conditions. Using a different AFM-based experimental approach at room temperature, Zhang et al. independently investigated the force-dependent dissociation of α4β1/VCAM-1 interactions [16].

The present study extends these earlier observations by addressing the functional regulation of receptor-mediated adhesion using several independent experimental approaches. Molecular specificity was supported by inhibition of either interaction partner using function-blocking antibodies directed against VLA-4 or VCAM-1. In addition, measurements performed on immobilized VCAM-1 Fc fusion protein indicated direct receptor-mediated binding, whereas immobilized ICAM-1 Fc fusion protein failed to support comparable interactions. Finally, a low-molecular-weight mannose-derived α4β1 integrin antagonist independently reproduced the marked reduction in adhesion events observed with antibody-mediated blockade. The agreement between these complementary approaches strengthens the conclusion that the measured rupture events predominantly represent specific VLA-4/VCAM-1 interactions.

A central finding of the present work is that VLA-4 inhibitory approaches primarily reduced the probability of detectable adhesion events rather than the magnitude of the remaining rupture forces. The characteristic single-bond force distribution remained remarkably stable despite antibody-mediated or pharmacological inhibition. This observation suggests that regulation of metastatic B16 melanoma cell adhesion occurs predominantly through modulation of receptor–ligand encounter probability, receptor accessibility, or receptor organization at the cell surface rather than through substantial alterations in the mechanical strength of individual VLA-4/VCAM-1 bonds.

An exception to this general pattern was observed after VCAM-1 blockade, which shifted the rupture-force distribution toward lower values (Figure 4b). Full-length seven-domain VCAM-1 contains two independent VLA-4-binding sites located in Ig-like domains 1 and 4, either of which can support VLA-4-dependent adhesion [17,18]. Differential or incomplete blockade of these sites could potentially contribute to the residual interactions around 30 pN. However, the domain specificity of the antibody and the VCAM-1 isoform expressed by bEnd.3 cells were not determined, and this interpretation therefore remains hypothetical.

Residual low-force interactions may reflect other adhesion receptors or nonspecific protein–protein interactions that remain after blockade of the VLA-4/VCAM-1 axis. Although B16 melanoma cells express high levels of CD44, they did not bind to its ligand, hyaluronic acid (HA) and extensive previous flow chamber experiments did not reveal detectable CD44-mediated rolling on hyaluronan [6]. In contrast, recent experiments with monocytoid and primary monocyte populations demonstrated CD44–HA-dependent rolling under conditions in which α4-integrin-dependent adhesion was not dominant [15]. This illustrates that weaker or otherwise subordinate adhesion pathways may become functionally detectable when dominant integrin-mediated interactions are absent or reduced. Therefore, a major contribution of CD44 appears unlikely. Nevertheless, we cannot completely exclude the possibility that a small subpopulation of functional CD44 molecules becomes detectable at the single-molecule level after VLA-4 blockade, thereby contributing to a limited number of residual low-force interactions.

This behavior differs fundamentally from classical leukocyte recruitment. In leukocytes and hematopoietic progenitor cells, chemokines can rapidly enhance VLA-4-mediated interactions with VCAM-1 through G protein-dependent signaling and integrin clustering [19,20]. During earlier studies performed in the same laboratory, chemokine activation of VLA-4 by CXCL12 (SDF-1) produced measurable increases in VLA-4-mediated binding forces on lymphoma cells, demonstrating that AFM-based force spectroscopy is capable of detecting genuine force increases associated with integrin activation [21]. In contrast, the present results indicate that metastatic B16 melanoma cells do not require such chemokine-dependent force enhancement to establish efficient vascular adhesion. Together with the previous flow-chamber observations, the AFM data are consistent with productive VLA-4/VCAM-1 bond formation being sufficient to stabilize cell attachment under shear, while the cellular determinants of bond-formation probability remain unresolved.

The present AFM data therefore provide the molecular counterpart to our recent flow-chamber observations. Whereas the flow experiments showed that VLA-4 is necessary and sufficient for both rolling and immediate firm arrest of metastatic B16 melanoma cells under physiological shear conditions without chemokine activation, the AFM experiments explain how this behavior can occur at the receptor level. Importantly, the physiological relevance of the receptor interactions detected by AFM is independently validated by the corresponding flow-chamber experiments performed under physiological shear conditions. Together, the two studies establish a direct functional link between receptor-specific single-molecule interactions and macroscopic tumor-cell adhesion under flow. This complementary combination of flow-chamber analysis and AFM-based single-molecule force spectroscopy provides experimental evidence across multiple biological scales, linking receptor-level mechanics directly to tumor-cell rolling and vascular arrest under physiological shear conditions. The observation that VLA-4 also mediates homotypic adhesion between B16 melanoma cells further broadens the biological significance of this receptor. Previous work indicated that VLA-4 expression may reduce dissemination from established primary tumors by promoting tumor-cell aggregation [22]. Conversely, VLA-4 has also been implicated in experimental melanoma metastasis and in melanoma-cell adhesion to and extravasation through endothelium under low-flow conditions [23,24]. Once tumor cells enter the circulation, the same receptor may facilitate efficient vascular arrest through interaction with endothelial VCAM-1 [6]. Thus, VLA-4 may exert context-dependent functions during different stages of the metastatic cascade, illustrating how a single adhesion receptor can contribute to apparently opposing aspects of tumor progression. Context-dependent effects of α4 integrins on distinct steps of tumor metastasis have also been observed in lymphoma models [25].

Because melanoma cell adhesion differs fundamentally from the classical leukocyte paradigm, the present AFM assay may also provide a useful platform for evaluating VLA-4-directed antibodies and small-molecule inhibitors. Comparative studies using melanoma cells and leukocytes could help determine whether different classes of VLA-4 inhibitors differentially affect pathological and physiological adhesion. Such analyses may be relevant not only for metastatic melanoma but also for inflammatory diseases such as multiple sclerosis, where therapeutic VLA-4 blockade is already established [26].

Several limitations should be acknowledged. The present experiments were performed using established murine cell lines under controlled in vitro conditions optimized for predominantly single-molecule measurements. Although these conditions allow direct analysis of receptor–ligand mechanics, additional adhesion receptors, endothelial activation states, and hemodynamic conditions may contribute to tumor-cell arrest in vivo.

The present study focuses on initial adhesion and does not address subsequent transendothelial migration. Furthermore, the molecular identity of the residual low-force interactions detected after VLA-4 blockade remains unresolved and warrants further investigation. Future studies should determine whether similar mechanisms operate in human melanoma cells and additional tumor entities expressing VLA-4. Such approaches may also be useful for studying functionally heterogeneous tumor-cell populations, including tumor cells with stem-like properties [27]. Receptor-level force measurements may also be applicable to circulating tumor cells (CTCs) from other malignancies. For example, intact CTCs and an unexpected hematogenous route of dissemination have been described in medulloblastoma, and AFM-based biophysical characterization has been proposed as a potential approach for studying such cells and their metastatic interactions [28]. Complementary live-cell imaging approaches, including fluorescent carbon dots for organelle targeting and real-time analysis of cellular responses, may provide additional dynamic cellular information alongside force-based measurements [29].

In conclusion, the present study provides the first direct functional extension of earlier AFM measurements by linking receptor-specific single-molecule force spectroscopy to the chemokine-independent rolling and arrest phenotype previously shown under physiological flow. Rather than altering the strength of individual receptor–ligand bonds, functional inhibition primarily decreases the probability of productive VLA-4/VCAM-1 bond formation. Together with our recent flow chamber study, these findings establish a direct functional link between receptor-specific single-molecule interactions, chemokine-independent tumor-cell rolling, and vascular arrest under physiological shear conditions.

## 5. Conclusions

AFM-based single-molecule force spectroscopy demonstrates discrete VLA-4/VCAM-1 receptor-level interactions contributing to B16 melanoma cell adhesion. Extending earlier AFM measurements of single VLA-4/VCAM-1 bonds, the present study shows that functional inhibition predominantly reduces the probability of adhesion events, while the characteristic rupture-force range is largely preserved. Together with the previous flow-chamber study, these findings provide molecular insight into VLA-4/VCAM-1 interactions underlying chemokine-independent tumor cell arrest and establish a link between integrin-mediated adhesion under shear flow and receptor-level interactions. This framework highlights the importance of integrin-dependent adhesion mechanisms in early metastatic dissemination.

## Data Availability

The data supporting the findings of this study are available from the corresponding author upon reasonable request. The original AFM data are retained in archived electronic and laboratory records.

## References

[1] Liotta L.A., Steeg P.S., Stetler-Stevenson W.G. (1991). Cancer metastasis and angiogenesis: An imbalance of positive and negative regulation. Cell.

[2] Butcher E.C. (1991). Leukocyte-endothelial cell recognition: Three (or more) steps to specificity and diversity. Cell.

[3] Springer T.A. (1994). Traffic signals for lymphocyte recirculation and leukocyte emigration: The multistep paradigm. Cell.

[4] Campbell J.J., Hedrick J., Zlotnik A., Siani M.A., Thompson D.A., Butcher E.C. (1998). Chemokines and the arrest of lymphocytes rolling under flow conditions. Science.

[5] Alon R., Kassner P.D., Carr M.W., Finger E.B., Hemler M.E., Springer T.A. (1995). The integrin VLA-4 supports tethering and rolling in flow on VCAM-1. J. Cell Biol..

[6] Eibl R.H. (2026). Chemokine-Independent VLA-4/VCAM-1-Mediated Rolling and Arrest of B16 Melanoma Cells Under Shear. Int. J. Mol. Sci..

[7] Muller D.J., Dufrene Y.F. (2008). Atomic force microscopy as a multifunctional molecular toolbox in nanobiotechnology. Nat. Nanotechnol..

[8] Eibl R.H., Benoit M. (2004). Molecular resolution of cell adhesion forces. IEE Proc. Nanobiotechnol..

[9] Benoit M., Gabriel D., Gerisch G., Gaub H.E. (2000). Discrete interactions in cell adhesion measured by single-molecule force spectroscopy. Nat. Cell Biol..

[10] Zhang X., Wojcikiewicz E., Moy V.T. (2002). Force spectroscopy of the leukocyte function-associated antigen-1/intercellular adhesion molecule-1 interaction. Biophys. J..

[11] Helenius J., Heisenberg C.P., Gaub H.E., Muller D.J. (2008). Single-cell force spectroscopy. J. Cell Sci..

[12] Boer J., Gottschling D., Schuster A., Holzmann B., Kessler H. (2001). Design, Synthesis, and Biological Evaluation of α_4_β_1_ Integrin Antagonists Based on β-D-Mannose as Rigid Scaffold. Angew. Chem. Int. Ed. Engl..

[13] Eibl R.H., Moy V.T. (2005). Atomic force microscopy measurements of protein-ligand interactions on living cells. Methods Mol. Biol..

[14] Florin E.L., Moy V.T., Gaub H.E. (1994). Adhesion forces between individual ligand-receptor pairs. Science.

[15] Hubbe M., Eibl R.H. (2026). CD44-Hyaluronan-Dependent Monocyte Rolling. Int. J. Mol. Sci..

[16] Zhang X., Craig S.E., Kirby H., Humphries M.J., Moy V.T. (2004). Molecular basis for the dynamic strength of the integrin α_4_β_1_/VCAM-1 interaction. Biophys. J..

[17] Vonderheide R.H., Tedder T.F., Springer T.A., Staunton D.E. (1994). Residues within a conserved amino acid motif of domains 1 and 4 of VCAM-1 are required for binding to VLA-4. J. Cell Biol..

[18] Osborn L., Vassallo C., Benjamin C.D. (1992). Activated endothelium binds lymphocytes through a novel binding site in the alternately spliced domain of vascular cell adhesion molecule-1. J. Exp. Med..

[19] Peled A., Grabovsky V., Habler L., Sandbank J., Arenzana-Seisdedos F., Petit I., Ben-Hur H., Lapidot T., Alon R. (1999). The chemokine SDF-1 stimulates integrin-mediated arrest of CD34(+) cells on vascular endothelium under shear flow. J. Clin. Investig..

[20] Grabovsky V., Feigelson S., Chen C., Bleijs D.A., Peled A., Cinamon G., Baleux F., Arenzana-Seisdedos F., Lapidot T., van Kooyk Y. (2000). Subsecond induction of α4 integrin clustering by immobilized chemokines stimulates leukocyte tethering and rolling on endothelial vascular cell adhesion molecule 1 under flow conditions. J. Exp. Med..

[21] Eibl R.H. (2004). Atomic force microscopy measurement of SDF-1 mediated affinity modulation of single VLA-4—VCAM-1 bonds. Immunology 2004: Cytokine Network, Regulatory Cells, Signaling and Apoptosis.

[22] Qian F., Vaux D.L., Weissman I.L. (1994). Expression of the integrin α4β1 on melanoma cells can inhibit the invasive stage of metastasis formation. Cell.

[23] Garofalo A., Chirivi R.G., Foglieni C., Pigott R., Mortarini R., Martin-Padura I., Anichini A., Gearing A.J., Sanchez-Madrid F., Dejana E. (1995). Involvement of the very late antigen 4 integrin on melanoma in interleukin 1-augmented experimental metastases. Cancer Res..

[24] Liang S., Dong C. (2008). Integrin VLA-4 enhances sialyl-Lewisx/a-negative melanoma adhesion to and extravasation through the endothelium under low flow conditions. Am. J. Physiol. Cell Physiol..

[25] Gosslar U., Jonas P., Luz A., Lifka A., Naor D., Hamann A., Holzmann B. (1996). Predominant role of alpha 4-integrins for distinct steps of lymphoma metastasis. Proc. Natl. Acad. Sci. USA.

[26] Miller D.H., Khan O.A., Sheremata W.A., Blumhardt L.D., Rice G.P., Libonati M.A., Willmer-Hulme A.J., Dalton C.M., Miszkiel K.A., O’Connor P.W. (2003). A controlled trial of natalizumab for relapsing multiple sclerosis. N. Engl. J. Med..

[27] Reya T., Morrison S.J., Clarke M.F., Weissman I.L. (2001). Stem cells, cancer, and cancer stem cells. Nature.

[28] Eibl R.H., Schneemann M. (2023). Medulloblastoma: From TP53 Mutations to Molecular Classification and Liquid Biopsy. Biology.

[29] Unnikrishnan B., Anand A., Lin C.-J., Lee C.-Y., Nain A., Srivastava P., Wu R.-S., Chu H.-W., Wang C.-Y., Shi R.-H. (2025). Exploring cellular dynamics: Engineered fluorescent carbon dots for organelle staining and cellular response analysis. Coord. Chem. Rev..

